# Biological Deciphering of the “Kidney Governing Bones” Theory in Traditional Chinese Medicine

**DOI:** 10.1155/2022/1685052

**Published:** 2022-03-29

**Authors:** Hanmin Zhu, Qi Liu, Wei Li, Shuming Huang, Bo Zhang, Yumei Wang

**Affiliations:** ^1^Hubei University of Arts and Science, HuBei, XiangYang 441053, China; ^2^Qiqihar Medical University, Heilongjiang, Qiqihar 161006, China; ^3^Heilongjiang University of Chinese Medicine, Heilongjiang, Harbin 150040, China

## Abstract

The description of the “kidney” was entirely different from modern medicine. In traditional Chinese medicine (TCM), the kidney was a functional concept regulating water metabolism, which was closely related to the urinary system, reproductive system, nervous system, endocrine, skeleton, hearing, metabolism, immunity, etc. In particular, the kidney in TCM plays an important regulatory role in the processes of growth, development, prime, aging, and reproduction. Hence, “Kidney Governing Bone” (KGB) was a classical theory in TCM, which hypothesized that the function of the kidney was responsible for bone health. However, the related modern physiological mechanisms of this TCM theory are unclear. This present paper proposed a new understanding and explored the biological basis of the KGB theory. After searching through plenty of reported literature, we discovered that the functions of the kidney in TCM were closely associated with the hypothalamic-pituitary-gonadal (HPG) axis in modern science. The physiological mechanism of the KGB was regulated by sex hormones and their receptors. This review deciphered the connotation of the KGB theory in modern medicine and further verified the scientificity of the basic TCM theory.

## 1. Introduction

In traditional Chinese medicine (TCM), the kidney was a functional concept regulating water metabolism which was closely related to the urinary system, reproductive system, nervous system, endocrine, skeleton, hearing, metabolism, immunity, etc. It plays an important regulatory role in the processes of growth, development, prime, aging, and reproduction. Centuries ago, TCM owned abundant unique theories, of which the “Kidney Governing Bones” (KGB) theory was a vital one. This theory has been applied in the treatment of bone diseases over thousands of years. In the meantime, kidney reinforcing prescriptions have been confirmed with admirable effect by TCM practices in China [1–3]. However, the modern biological deciphering of the KGB theory remains unclear up to now.

Various hypotheses about mechanism of the KGB theory were proposed successfully. The main viewpoints were summarized as follows: (1) the kidney could affect the absorption of vitamin D; (2) the kidney could regulate the metabolism of trace elements such as calcium and phosphorus; (3) the kidney could regulate the secretion and metabolism of growth hormone; (4) the kidney could affect the OPG-RANKL-RANK signaling pathway; (5) the kidney could regulate the neuroendocrine-immune network (N-E-IN) [4, 5]. In brief, the kidney in TCM could regulate the processes of bone growth and metabolism through a variety of pathways. Sufficient understanding of the KGB theory could provide new insights for treating bone diseases in clinical trials. To our pleasure, plenty of physiology experimental results verified the scientificity of the abovementioned viewpoints.

Recently, studies have indicated that sex hormones play indispensable role in skeletal size, skeletal shape, and skeletal homeostasis during growth [6]. It was well known that the hypothalamic-pituitary-gonadal (HPG) axis carried out functions through sex hormones. Therefore, functions of the kidney in TCM were primarily related to functions of the HPG axis [7]. This article aimed to explore a modern scientific explanation of the KGB theory based on currently available studies on bone.

## 2. Definition of the Kidney in TCM

**Figure 1:**
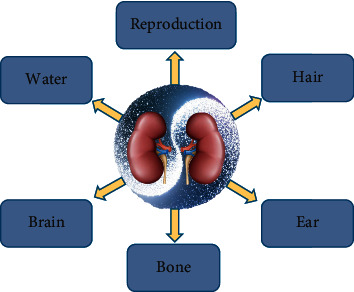
“Kidney governing bones” theory in TCM.

TCM described “kidneys” as a pair of organs located in the lumbar region, as the same in modern anatomy. However, the functions of the kidney in TCM were not identical to those in modern medicine. In TCM, the functions of the kidney include storage of essence, governance of water, bone protection, auditory regulation, reproduction promotion, and brain communication, as shown in Figure 1. In particular, according to an ancient book named *Huang Di Nei Jing Su Wen*, essence stored in the kidney was vital for human life. Therefore, the kidney in TCM is a functional concept, which is quite different from the definition in modern medicine.

## 3. The Correlation of the Function of the Kidney in TCM and the HPG Axis Biological Action in Modern Physiology

According to TCM, the kidney governs the reproductive function of humans. Both the maturity of sexual organs and the maintenance of sexual function are associated with the storage of essence in the kidney. According to an ancient TCM book titled *Internal Classic Suwen*, a woman's kidney energy became vigorous at the age of 7 and Tiangui arrived at 14, after which the conception vessel and thoroughfare vessel became vigorous and the menstruation appeared, and the woman was able to conceive a baby. At the age of 49, as the conception vessel and thoroughfare vessel became deficient, the menstruation stopped, then the woman became physically feeble and was no longer able to conceive a baby. However, men's kidneys become exuberant at the age of 8, then the kidney energy becomes prosperous at 16. After Tiangui arrived, the kidney essence became vigorous, and man was able to have an offspring. At the age of 64, as the kidney essence becomes deficient, the man becomes physically feeble and finally loses the ability to have a baby. Reproductive function depended on the conception vessel and thoroughfare vessel. Furthermore, the prosperity or decline of the conception vessel and thoroughfare vessel depended on Tiangui maturity and failure; however, Tiangui was derived from the essence of the kidney in TCM. Thus, a functional unit named “kidney-Tiangui-Conception vessel/thoroughfare vessel-Reproductive function” axis was built.

In modern physiology, the hypothalamus, a part of the hypothalamus-pituitary functional unit, is closely related to neuromodulation and humoral regulation. Peptidergic neurons, which are distributed in the pituitary gland's arcuate nucleus of the hypothalamus, could secrete regulatory peptides. As a regulating peptide, gonadotropin-releasing hormone could regulate the release of gonadotropin by the pituitary gland through binding to a target membrane receptor. Furthermore, gonadotropin could regulate the secretion of sex hormones in gonads. This functional unit was widely known as the HPG axis. The HPG axis was involved in the process of sexual maturity. With the progression of age, the functions of the HPG axis descended consequently, and the reproductive function declined. Evidently, the reproductive function of the kidney in TCM was in fact the function of the HPG axis according to modern physiology.

## 4. Role of the Kidney/HPG Axis in the Maintenance of Bone Function and Bone Growth

### 4.1. Low Level of Sex Hormone Resulted in a High Incidence of Bone Diseases

Bone mass depends on the coordinated activities of bone-forming osteoblasts and bone-resorbing osteoclasts. Furthermore, the activity of osteoblasts and osteoclasts is controlled by a variety of hormones and cytokines [8]. Sex hormone was crucial to maintain bone mass; the lack of estrogen or testosterone resulted in a high incidence of bone diseases.

A large number of animal experimental studies indicated that the deficiency of sex hormones led to osteoporosis, which involved a decline of bone mass, changes of bone tissue microstructure, reduction of bone mineral density, and a loss of bone strength [9, 10]. These characteristics were observed in both male and female castrated animals [11]. Ovariectomized animals including rats [12], mice [13], and even sheep [14] have been used as disease models studying the generation and treatment of osteoporosis. In male animals, orchiectomy has been reported to reduce bone mineral density and bone intensity. Male animals that underwent orchiectomy were used as osteoporosis models [15–18].

In clinical practice, it was well known that sex hormones were the most important factor involved in the maintenance of bone mass. Deficiency of sex hormone was considered as a major cause of bone loss in osteoporosis patients [19–21]. An epidemiological study including 25,544 prostate cancer patients from 2004 to 2012 in New Zealand confirmed the association between androgen deprivation therapy (ADT) and fracture risk, demonstrating that ADT significantly increased the risk of bone diseases [22]. Another large cohort study investigated the influence of sexual hormone levels on bone diseases in 1,249 testicular cancer survivors and finally confirmed that testosterone deficiency was closely associated with bone diseases [23, 24]. In addition to some pathological factors, there are many drugs that can affect the hypothalamic-pituitary-gonadal (HPG) axis and then cause low bone mineral density, such as opiates and hormones [24–26].

### 4.2. Sex Hormones Acted on Bone as Growth Factors

Many factors are involved in the regulation of bone maturation, such as growth hormone, insulin-like growth factors, gonadal steroids, and other factors [27, 28]. Among these, sex hormones were currently considered as the most important factor associated with bone growth. Previous studies indicated that both testosterone and estradiol positively regulated the growth of the skeleton during the process of individual development, especially in childhood and adolescence. The levels were closely associated with areal bone mineral density and volumetric bone mineral density [29–31]. Reportedly, a decrease in estrogen level was observed in adolescent and young female athletes undergoing intense training regimens, and this decline was accompanied by a deficiency in peak bone mass [32]. Furthermore, lower bone mineral density was also observed in young men with hypogonadism in clinical studies [33]. Testosterone treatment has a significant improvement in lumbar spine and hip BMD in men with testosterone deficiency syndrome [34]. Studies showed that estrogen stimulated cancellous bone formation in both female and male animals. Estradiol increased the proportion of cancellous bone surfaces undergoing mineralization and mineral apposition rate in male mice [35], and estradiol increased areal bone mineral density, trabecular bone volume/tissue volume in vertebrae, and cortical thickness in the axial skeleton mediated by estrogen receptor-*α* in ovariectomized female mice [36, 37]. Furthermore, testosterone was also found to be essential for the formation of bone and maintenance of its structure; it increased the density of periosteal bone and trabecular bone via the androgen receptor in male mice [38]. Another study found that testosterone increased bone mineral density in the femur and spine and improved bone architecture (increased bone volume fraction, trabecular number, thickness, and connectivity density) in ovariectomized mice [39].

### 4.3. Involvement of Sex Hormones in Bone Metabolism

Bone metabolism was a complex process, the maintenance of bone structure and function was carried out by bone cells. In bone remodeling process, osteoclasts and osteoblasts play an important role [40]. Studies revealed that sex hormones were vital for bone metabolism [41]. Estrogen reduced the reabsorption of bone by inhibiting osteoclast activity [42] and enhanced the synthesis by increasing the number of osteoblasts by inhibiting apoptosis [43], decreasing oxidative stress [44], and reducing nuclear factor-*κ*B (NF-*κ*B) activity [45]. Hence, estrogen regulated bone metabolic process by interfering with remodeling and reabsorption [46, 47]. It was especially worth clarifying that estrogen was involved in the regulation of bone metabolism in both women and men [48]. However, the effects of testosterone on bone health were not well defined. Recently, studies showed that serum testosterone levels were associated with the risk of osteoporotic fracture in old men [49–51].

### 4.4. Involvement of Sex Hormones in Bone Metabolism Signaling Pathways

Plenty of cellular signaling pathways act as various networks by regulating or restricting each other, maintaining bone homeostasis [52]. Bone morphogenetic protein (BMP)/Smads, Wnt/*β*-catenin, and osteoprotegerin (OPG)/RANKL/RANK are considered as key signaling pathways that play important roles in bone metabolism [53, 54]. The BMP/Smads and Wnt/*β*-catenin signaling pathways are mainly responsible for bone formation, while the OPG/receptor activator of NF-*κ*B ligand (RANKL)/RANK is mainly responsible for bone reabsorption [55]. Sex hormone affect bone metabolism by regulating the signaling pathways through a variety of mechanisms, such as regulating the expression of genes and affecting receptors [56, 57].

### 4.5. Involvement of Kidney's Endocrine Function in Bone Metabolism

The kidney is not only an anatomical organ, but also a complex endocrine organ. Many components or hormones secreted by the kidney are involved in the regulation of bone metabolism. Erythropoietin (EPO) is a key regulator in bone remodeling produced in the kidney during adult life. Multipotent MSCs undergo EPO induced osteogenic transdifferentiation via the activation of EPO-R signaling pathways, leading to bone remodeling [58]. 1,25-dihydroxy-vitamin D_3_, the active component of vitamin D, was produced in the kidney and mainly participated in bone formation by regulating calcium and phosphorus metabolism. While recent research studies suggested that vitamin D in bone appeared to have negative or positive roles depending on physiological and pathological circumstances, suggesting that vitamin D played pleiotropic role in bone metabolism [59]. Klotho expressed in the kidney coparticipated in the regulation of bone metabolism with FGF23 [60]. BMP was a powerful bone-inducing stimulator of bone formation and the cloud promoted the process of bone regeneration by promoting osteoblast precursors and osteoblast terminal differentiation from progenitor mesenchymal cells and bone progenitor cells. BMP-7 is an important member of the bone morphogenetic protein family that can be secreted by the kidney and promotes bone formation [61].

### 4.6. The Relationship of Neuroendocrine-Immune Network and the “KGB” Theory

The neuroendocrine-immune network (N-E-IN) suggested that there may be a common set of chemical information molecules and receptors among the neural, endocrine, and immune system. The intricate and complex interactions among these systems form a multidimensional network maintaining homeostasis and improving body function [62]. Estrogen affects the regulatory functions of the N-E-IN through a variety of ways [63]. The serotonin molecule is a classic neuroendocrine hormone. The serotonin worked on bone via two ways: (1) serotonin produced from peripheral acted as a hormone to inhibit bone formation, and (2) serotonin produced in the brain acted as a neurotransmitter exerting positive effect on bone mass by enhancing bone formation and limiting bone resorption [64]. Orexin is a critical neuropeptide that bidirectionally regulated skeletal homeostasis through positive and negative action. The functions depended on the distribution of orexin the blinked receptor. Orexin receptor 2 in the brain enhanced bone formation while orexin receptor 1 in the bone suppressed bone formation [65]. Osteocytes could secret neuropeptide Y under the control of the autonomic nervous system. Then, neuropeptide Y regulated bone marrow mesenchymal stem cells towards adipocytes rather than osteoblasts and finally led to bone loss and multiple bone diseases [66]. Substance P (SP) is an endogenous neuropeptide. SP ameliorated chronic inflammation by promoting the immune system and rejuvenating stem cells to promote the regeneration of osteoblasts [67]. Bone metabolism in the central nervous system is mainly regulated by the ventral hypothalamus. Galanin, a hypothalamic regulatory peptide, is an important regulatory factor [68]. However, the GABAergic neural circuit in the ventromedial hypothalamus can trigger bone loss without stressors [69]. To sum up, N-E-IN and “KGB” have a close relationship with each other. The mechanisms of bone homeostasis with “KBG” are shown in Table 1.

## 5. Kidney Deficiency Resulted in Bone Diseases

As bone is governed by the kidney in TCM, the sufficiency of the kidney essence is considered to be closely associated with the growth and function of bone. According to the ancient Chinese medical book named *Huangdi Neijing*, the kidney stores essence, essence generates marrow, marrow produces bone, and therefore, bone is governed by the kidney. With the progression of age, the kidney essence decreases gradually, and the individual becomes susceptible to bone diseases. Clinical studies reported that most patients with osteoporosis or lumbar intervertebral disc degeneration show symptoms of kidney deficiency. A high incidence rate of bone disease was observed in patients with impaired kidney function [70]. Some scholars investigated the association between kidney deficiency and bone mineral density using epidemiological methods, and indicated that bone mass loss and the incidence of osteoporosis in subjects with kidney deficiency were obviously higher than those in controls [71]. Furthermore, animal experiments confirmed that kidney deficiency affected the growth and condition of bones. It was reported that decreased collagen synthesis resulted in the degeneration of cervical vertebra, lumbar intervertebral disc, and articular cartilage of the knee in several kidney deficiency rat models [72].

## 6. Kidney Tonifying Prescriptions Showed Protection via Sex Hormones/Receptors

The kidney essence is especially vital for bones in TCM. Many bone diseases are caused by the insufficiency of kidney essence, and kidney tonifying prescriptions have ideal effects on some chronic diseases of the bone, as shown in Table 2. Osteoporosis is a metabolic bone disease that mainly occur in old people. Clinical research articles on the effects of kidney tonifying prescriptions on bone disease showed that many kidney tonifying prescriptions, such as Zuogui-wan [73], Yougui-wan [74, 75], and Liuweidihuang Pill [76, 77], [78–81] were effective in treating osteoporosis. The mechanisms by which kidney tonifying prescriptions treat bone diseases are complex. However, recently, some studies revealed that common pathways are increasing the effects of sex hormones/receptors or showing sex hormone-like action [82].

## 7. Summary

**Figure 2:**
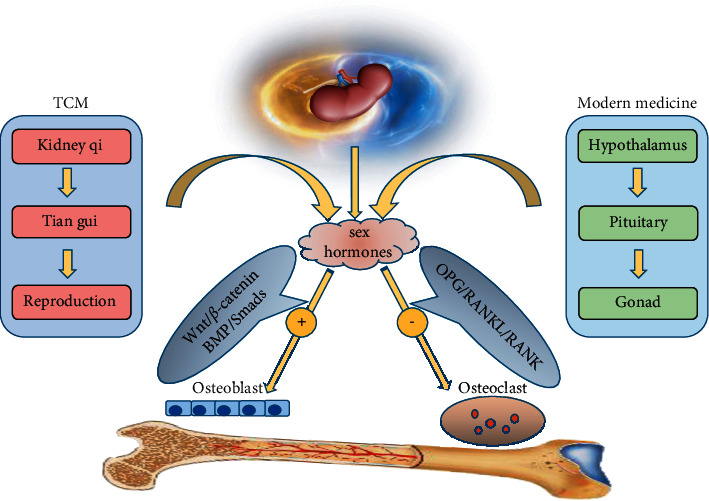
Biological deciphering of the “Kidney governing bones” theory in traditional Chinese medicine.

Summing up the above study, the functions of the kidney in TCM were in accordance with the functions of the HPG axis in modern physiology. The abovementioned content offered biological evidence of the KGB theory. In this paper, we clarified that the kidney in TCM is a functional concept, and the functions of the “Kidney-Tian gui-vital essence-reproductive function” axis in TCM and the HPG axis of modern science are closely similar. Sex hormone, the executors of HPG axis function, played a critical role in the maintenance of bone. Kidney deficiency or sex-hormone deficiency results in a high risk of bone diseases. Protective effects of kidney tonifying prescriptions in TCM were mediated by the sex hormone-like actions. In conclusion, we proposed a new explanation for the biological essence of the KGB theory in TCM, illustrating that the kidney mainlyregulats the actions of sex hormones or receptors that govern the bone. The biological deciphering of KGB theory was summarized as Figure 2.

## Figures and Tables

**Table 1:** Mechanisms of bone homeostasis with the “KGB” theory.

Pathways of regulating bone homeostasis	Main mechanisms	References
As growth factors	Increasing bone mineral density, promoting bone formation, and maintaining bone structure by sex hormones/their receptors	[27–39, 69]

Regulate bone metabolism	Inhibiting osteoclast activity and increasing the number of osteoblasts by sex hormones	[41–51]

Regulate signaling pathways	Regulating the signaling pathway, such as BMP/Smads, Wnt/*β*-catenin, and OPG/RANKL/RANK by sex hormones	[53–57]

Affect endocrine function	Many components or hormones secreted by the kidney-EPO, 1,25-dihydroxy-vitamin D_3_, klotho, BMP-7, and exosomes were involved in the regulation of bone metabolism	[58–61]

Affect N-E-IN	Neuropeptide derived from the N-E-IN, such as serotonin, orexin, neuropeptide Y, substance P, galanin, and GABAergic, are involved in maintaining bone homeostasis	[64–68]

**Table 2:** Kidney tonifying prescriptions via sex hormones/receptors.

Tonifying kidney prescriptions	Composition of TCM	References
Zuogui-Wan	Praeparata, dogwood, yam, deer-horn glue, tortoise-plastron glue, *Fructus lycii*, dodder seed, and Radix Achyranthis Bidentatae	[73]
Yougui-Wan	Praeparata, dogwood, yam, deer-horn glue, *Fructus lycii*, dodder seed, cinnamon, angelica, monkshood, and *Eucommia ulmoides*	[74, 75]
Liuweidihuang pill	*Rehmannia* root, *Cornus officinalis* siebold, Chinese yam, *Alisma plantago-aquatica* subsp., *Paeonia suffruticosa*, and *Poria cocos*	[76, 77]
Xian-Ling-Gu-Bao capsule	Herba epimedii, Radix Dipsaci, *Fructus psoraleae*, Rhizoma anemarrahenae, Radix rehmanniae, and Radix salviae miltiorrhizae	[78, 79]
AS1350	Cornu cervi pantotrichum, cinnamomi cortex, Radix *Rehmanniae*, Schisandra chinensis fructus, Barbary wolfberry fruit, Semen juglandis, Arillus longan, and Fructus ziziphi jujubae	[80]
Nanshi oral liquid	Polygoni multiflori radix, Corni fructus, Morindae officinalis radix, Rosae laevigatae fructus, Jujubae fructus, Juglandis semen, and Longan arillus	[81]
*Morinda* officinalis capsule	*Morinda*	[82]

## Data Availability

The data supporting this review came from previously reported studies which have been cited.
